# O.2.1-2 How do social prescribing interventions for physical activity work from a complex intervention research perspective: a narrative review on mechanisms of change

**DOI:** 10.1093/eurpub/ckad133.107

**Published:** 2023-09-11

**Authors:** Julie Sandell Jacobsen, Rasmus Oestergaard Nielsen, Nanna Holt Jessen, Lene Gissel Rasmussen, Halfdan Thorsø Skjerning, Dea Kejlberg Jensen, Solvej Videbæk Bueno, Per Kallestrup, Jeanette Reffstrup Christensen, Knud Ryom

**Affiliations:** Research Unit for General Practice, Aarhus, Denmark; Research Centre for Health and Welfare Technology, Programme for Rehabilitation, VIA University College, Denmark; Research Unit for General Practice, Aarhus, Denmark; Department of Public Health, Aarhus University, Denmark; Research Unit for General Practice, Aarhus, Denmark; Research Unit for General Practice, Aarhus, Denmark; Research Unit for General Practice, Aarhus, Denmark; Research Unit for General Practice, Aarhus, Denmark; Research Unit for General Practice, Aarhus, Denmark; Research Unit for General Practice, Aarhus, Denmark; Department of Public Health, Aarhus University, Denmark; Research Unit for General Practice, Aarhus, Denmark; Research Unit of General Practice, Department of Public Health, University of Southern Denmark, Odense, Denmark; Research Unit of User Perspectives and Community-Based Interventions, Department of Public Health, University of Southern Denmark, Odense, Denmark; jsaj@via.dk; Department of Public Health, Aarhus University, Denmark

## Abstract

**Purpose:**

Social prescribing (SP) interventions for physical activity (PA) remain to reveal consistent findings on health outcomes, partly because suboptimal research methods have been applied. The purpose of this study was to synthesise the behaviour-related mechanisms and socio-economic considerations of SP interventions for PA based on a narrative literature review. The synthesis was then used to develop a logic model inspired by the methodology from complex intervention research, illustrating how these interventions may improve health outcomes.

**Methods:**

A narrative review was performed using Medline, Embase and PsycINFO to identify scientific literature on SP interventions for PA. The literature search was executed on February 20-21, 2023. Inclusion criteria were; studies investigating person-centred mechanisms and/or system-based mechanisms. Any population was eligible, and no source restrictions were applied. Findings were synthesised and illustrated in a comprehensive logic model on SP interventions for PA.

**Results:**

A total of 340 studies were identified from the electronic literature search, and 11 studies met the inclusion criteria. Person-centred mechanisms for successful SP intervention were identified as: (1) Integrating medical and social needs in the referral process; (2) Trusted and informed relationships in the linking process; and (3) Meaningful relationships in a non-stigmatising setting in the engagement process. System-based mechanisms for successful SP intervention were identified as the need to fully integrate the SP interventions in the General Practice and community organisations. In addition, successful SP intervention was, among other findings, explained through emotional buy-in from all SP agents and clients.

**Conclusion:**

The present study display mechanisms of SP interventions for PA, suggesting that they should include socio-economic considerations and preferably system thinking. We also urge for more complex intervention thinking in developing and evaluating SP interventions for PA to clarify the mechanisms of what works for whom and under which circumstances.

**Support/Funding Source:**

No funding source was applied for this study.

